# The Construction and Analysis of ceRNA Network and Patterns of Immune Infiltration in Mesothelioma With Bone Metastasis

**DOI:** 10.3389/fbioe.2019.00257

**Published:** 2019-10-18

**Authors:** Runzhi Huang, Jiawen Wu, Zixuan Zheng, Guanghua Wang, Dianwen Song, Penghui Yan, Huabin Yin, Peng Hu, Xiaolong Zhu, Haiyun Wang, Qi Lv, Tong Meng, Zongqiang Huang, Jie Zhang

**Affiliations:** ^1^Shanghai First Maternity and Infant Hospital, Tongji University School of Medicine, Shanghai, China; ^2^Division of Spine, Department of Orthopedics, Tongji Hospital Affiliated to Tongji University School of Medicine, Shanghai, China; ^3^Tongji University School of Medicine, Tongji University, Shanghai, China; ^4^Department of Orthopaedics, The First Affiliated Hospital of Zhengzhou University, Zhengzhou, China; ^5^Department of Orthopedics, School of Medicine, Shanghai General Hospital, Shanghai Jiaotong University, Shanghai, China; ^6^Tongji University School of Life Sciences and Technology, Shanghai, China

**Keywords:** mesothelioma, bone metastasis, ceRNA network, immune infiltration, prognosis, nomogram

## Abstract

**Background:** Mesothelioma is a rare and aggressive tumor. Bone metastasis often occurs in the later stages of this disease along with poor quality of life. Thus, it is important to explore the tumorigenesis and bone metastasis mechanism of invasive mesothelioma. For this purpose, we established two nomograms based on tumor-infiltrating immune cells and ceRNA networks to describe the molecular immunity and the clinical prediction of mesothelioma patients with bone metastasis.

**Method:** The expression profiles of mRNAs, lncRNAs, and miRNAs of 87 primary mesotheliomas were obtained from the TCGA database; there were four patients with bone metastasis and 83 patients without. We constructed a ceRNAs network based on the differentially expressed RNAs between mesothelioma and bone metastasis. CIBERSORT was used to distinguish 22 immune cell types from the tumor transcriptomes. Kaplan–Meier survival analysis and the Cox proportional hazards model were used to evaluate the prognostic value of each factor. Prognosis-associated immune cells and ceRNAs were applied to establish prediction nomograms. The receiver operating characteristic curves (ROC) and calibration curves were utilized to assess the discrimination and accuracy of the nomogram.

**Results:** Differential analysis revealed that 20 lncRNAs, 15 miRNAs, and 230 mRNAs were significantly different in mesothelioma samples vs. bone metastasis samples. We constructed the ceRNA network to include 10 protein-coding mRNAs, 8 lncRNAs, and 10 miRNAs. Nine of 28 ceRNAs were found to be significant in the Kaplan–Meier analysis. Out of the 22 cell types, the fraction of dendritic cells resting (*P* = 0.018) was significantly different between the bone metastasis group and the non-bone metastasis group. The ROC and the calibration curves, based on ceRNA networks and tumor-infiltrating immune cells, respectively, suggested acceptable accuracy (AUC of 3-year survival: 0.827, AUC of 5-year survival: 0.840; AUC of 3-year survival: 0.730; AUC of 5-year survival: 0.753). Notably, based on the co-expression patterns between ceRNAs and Immune cells, we found that the hsa-miR-582-5p, CASP9, dendritic cells resting, ANIX2, T cells CD8, and T cells CD4 memory resting might be associated with the mesothelioma bone metastasis.

**Conclusion:** Based on ceRNA networks and patterns of immune infiltration, our study provided a valid bioinformatics basis in order to explore the molecular mechanism and predict the possibility of mesothelioma bone metastasis.

## Introduction

Mesothelioma (MESO) is a rare and aggressive tumor with male predominance (Musk et al., 2017). It is derived from mesothelial cells existing in various organs. The incidence is higher in men rather than women, with the major cause being asbestos exposure (Dalsgaard et al., 2019; van Gerwen et al., 2019). Patients with mesothelioma do not have specific symptoms, which makes early diagnosis difficult. Thus, many mesothelioma patients have distant metastases when the primary tumor is discovered. Apart from pleural, mesothelioma can develop in many other serosal surfaces, including the peritoneum, liver capsule, pericardium, and vagina tunica. Distant metastases are located mostly from local drainage lymph nodes to the serosal surface of multiple organs, such as the lungs, brain, liver, and bone (King et al., 1997).

Traditionally, treatment strategies for mesothelioma are determined by the stage of cancer and the patients' physical condition. Cancer-directed surgery in early-stage mesothelioma can clearly improve survival (Kim et al., 2019). Radiotherapy and chemotherapy are applied to patients individually or are combined with surgery to prevent local recurrence. However, these treatments cannot improve survival dramatically, and no standard second-line therapy can be selected (Vogelzang et al., 2003; Infante et al., 2016; Trovo et al., 2019). Recently, immunotherapy is widely used for many kinds of tumors, mesothelioma included, and immunotherapy does show apparent survival benefits for mesothelioma patients (Brower, 2016). However, it is difficult to predict the benefits of this therapeutic method. Additionally, there is no prediction model for patients with mesothelioma that can be used to predict the possibility of distant bone metastases (Bertoglio et al., 2017).

In this study, we try to solve this issue and explore the molecular mechanism of mesothelioma bone metastasis. As both the competing endogenous RNA (ceRNA) network and immune cell subtypes may predict the prognosis and bone metastasis, we applied the CIBERSORT algorithm to gene expression profiles acquired from the cancer genome atlas (TCGA) to evaluate the cell fraction and establish a ceRNA network. Then, prognosis-associated immune cells and ceRNAs were applied to establish prediction nomograms. Moreover, we assessed the connection between immune cells and ceRNA networks to provide a bioinformatics basis for the discovery of possible molecular pathways.

## Materials and Methods

### Data Collection and Differential Gene Expression Analysis

Expression profiles of the primary mesothelioma and of bone metastasis were downloaded from TCGA's (https://tcga-data.nci.nih.gov/tcga/) database, including mRNA, lncRNA, and miRNA. We collected both HTseq-count and Fragments per kilobase of exon per million reads mapped (FPKM) profiles of 87 samples, comprising 83 primary mesotheliomas without bone metastasis and four primary tumors with bone metastasis. Demographic information and the survival endpoints of patients were also retrieved. After filtering non-mesothelioma specific expression genes (no expression was detected in neither the experimental group nor the control group), differences in the expression of each RNA between mesothelioma and bone metastasis were analyzed using the DEseq2. With a false discovery rate (FDR) adjusted *P* value < 0.05, the log(fold-change) > 1.0 or < −1.0 was defined as a downregulated or upregulated gene, respectively. The relevant data provided by TCGA are publicly available.

### Construction of the ceRNA Network

Before primary statistical analysis, the miRNA–mRNA interaction information based on experimental verification was predicted using the miRTarBase (http://mirtarbase.mbc.nctu.edu.tw/) (Chou et al., 2018), while the lncbase v.2 Experimental Module (http://carolina.imis.athena-innovation.gr/diana_tools/web/index.php?r=lncbasev2%2Findex-experimental) was used to predict lncRNA–miRNA interaction (Paraskevopoulou et al., 2016). Then, miRNAs regulated for both lncRNAs and mRNAs, showing significant results in hypergeometric testing and correlation analysis, were selected for visualization of the ceRNA network using Cytoscape v.3.5.1 (Shannon et al., 2003).

### Survival Analysis and Nomograms of Key Members in the ceRNA Network

We used Kaplan–Meier survival analysis curves and the Cox proportional hazards model to evaluate the prognostic value of all biomarkers. All significant biomarkers were integrated into the Cox regression model and the Lasso regression was employed to ensure that the multivariate models were not overfitting. Eventually, we built a nomogram based on the multifactor models to predict the prognosis of patients with mesothelioma. The receiver operating characteristic curves (ROC) and calibration curves were utilized to assess the discrimination and accuracy of the nomogram.

### CIBERSORT Estimation

To estimate the proportion of infiltrating immune cells, standard annotated gene expression data were uploaded to the CIBERSORT web portal (http://cibersort.stanford.edu/), with LM22 signature and 1,000 permutation applied to the algorithm. Only cases with CIBERSORT *P* < 0.05 were considered eligible for subsequent Kaplan-Meier analysis.

### Survival Analysis and Nomograms of Key Members of the Immune Cells

We used Kaplan–Meier survival analysis and Cox regression to detect the prognosis-associated cell types. The Wilcoxon rank-sum test was used to evaluate the association between cell subtypes and metastasis, with the outcome determined as the TNM stage. Immune cells showing significant association with prognosis in the initial Cox model were selected to build the nomogram. The receiver operating characteristic (ROC) curve was used to assess the sensitivity and specificity of the diagnostic and prognostic model, and these were quantified by the area under the ROC curve (AUC). The predictive accuracy of the nomogram was assessed by calibration curve and the concordance index (C-index). The relationships among 22 immune cells and between ceRNAs and immune cells were calculated using Pearson correlation coefficients.

Only the two-sided *P* value < 0.05 was considered to be of statistical significance. All statistical analysis was implemented with R version 3.5.1 software (Institute for Statistics and Mathematics, Vienna, Austria; www.r-project.org) [Package: GDCRNATools (Li et al., 2018), edgeR, ggplot2, rms, glmnet, preprocessCore, survminer, timeROC].

### Multidimensional Validation

**Figure 1:**
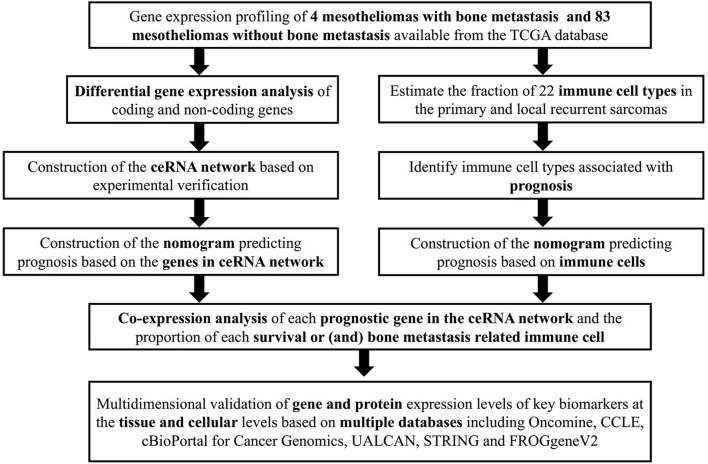
The flow chart of the analytical process.

To minimize bias caused by small study cohorts, multiple databases were used to explore the gene and protein expression levels of key biomarkers at tissue and cellular levels on the Oncomine (Contributors, 2006), cBioPortal for Cancer Genomics (Cerami et al., 2012; Gao et al., 2013), UALCAN (Chandrashekar et al., 2017), STRING (Szklarczyk et al., 2019), PROGgeneV2 (Goswami and Nakshatri, 2014) databases. The whole analytical process of this study is shown in Figure 1.

## Results

### Identification of Significant Differential Genes

**Figure 2:**
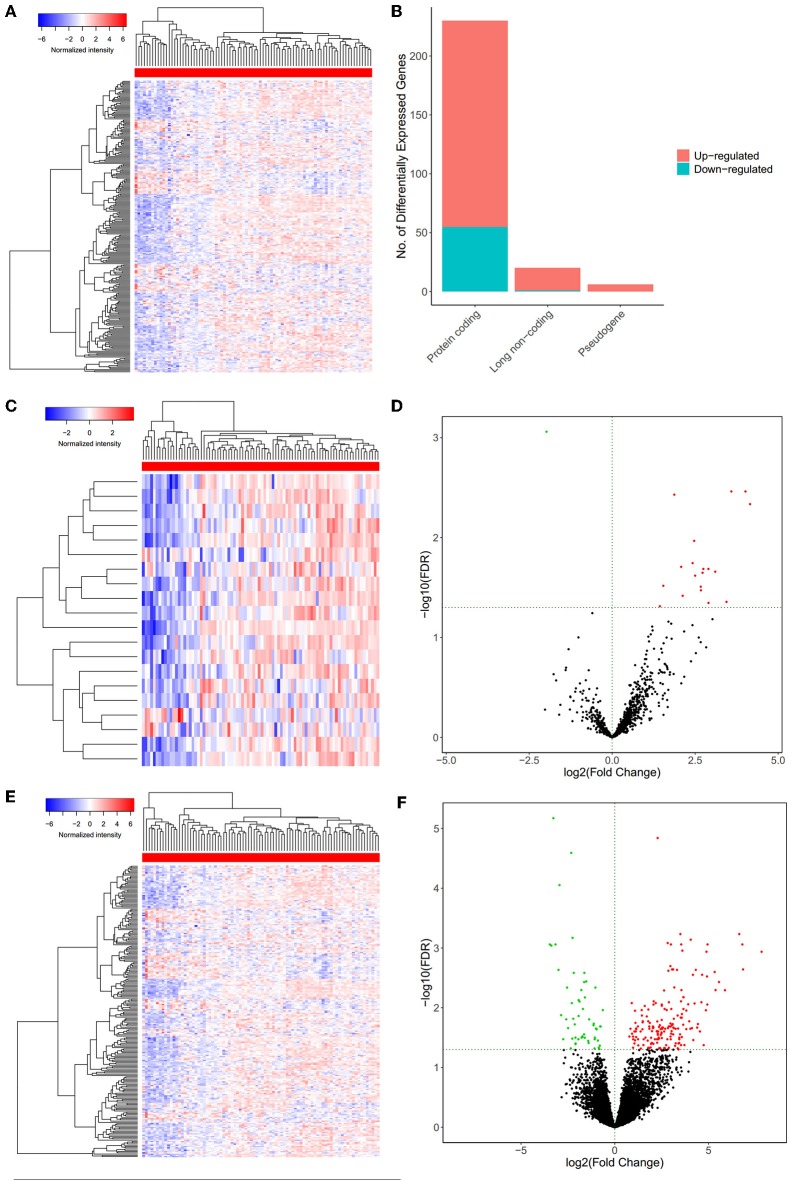
The differentially expressed messenger RNAs (mRNAs) between primary mesothelioma and bone metastasis mesothelioma. The heatmap of differentially expressed mRNAs between primary mesothelioma and bone metastasis **(A)**; the composition of differentially expressed genes **(B)**; the heatmap **(C);** and the volcano Plot **(D)** of differentially expressed protein-coding genes between primary mesothelioma and bone metastasis; the heatmap **(E);** and the volcano Plot **(F)** of differentially lncRNAs between primary mesothelioma and bone metastasis.

We used the log(fold-change) > 1.0 or < −1.0 and FDR <0.05 as cutoffs to identify differential RNA profiles. The baseline characteristics of all the patients available from the TCGA are described in Table S1. In a total of 14,447 lncRNAs, 2,588 miRNAs and 19,660 mRNAs from the TCGA database (Figures 2A,B), there are 15 differentially expressed miRNAs (2 downregulated and 13 upregulated). Figure 2 shows 230 differentially expressed protein-coding genes (55 downregulated and 175 upregulated) (Figures 2E,F) and 20 differentially expressed lncRNAs (19 downregulated and 1 upregulated) (Figures 2C,D).

### The Construction of the ceRNA Network and Survival Analysis

**Figure 3:**
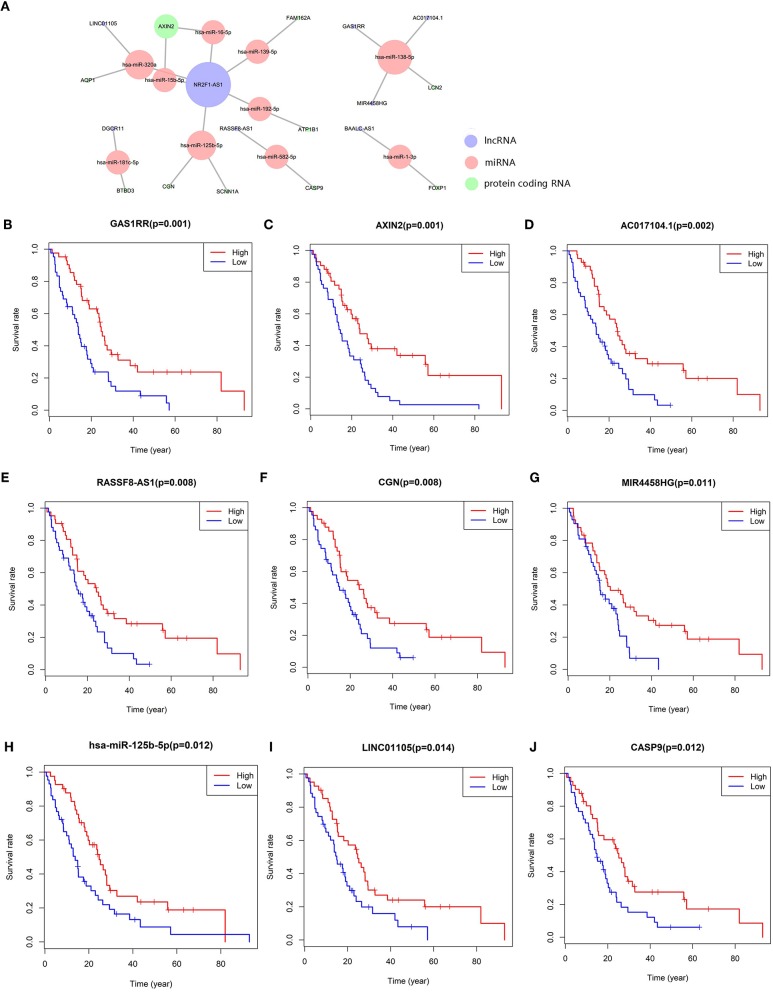
The meso-bone metastasis related ceRNA network **(A)**; the Kaplan–Meier survival curves of the key members of the ceRNA network: GAS1RR **(B)**, AXIN2 **(C)**, AC017104.1 **(D)**, RASSF8-AS1 **(E)**, CGN **(F)**, MIR4458HG **(G)**, hsa-miR-125b-5p **(H)**, LINC01105 **(I)**, and CASP9 **(J)**.

**Figure 4:**
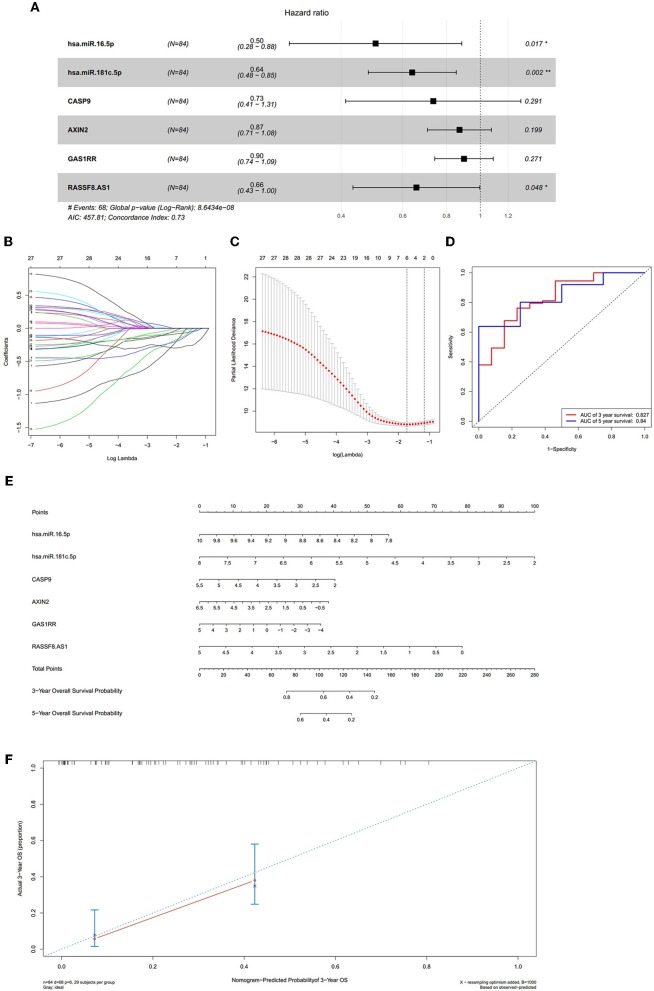
The results of the multivariate Cox regression **(A)**, the results of the Lasso regression **(B,C)**; the ROC curves **(D)**; the nomogram **(E)**; the discrimination of nomogram **(F)**. The results of the Lasso regression **(B,C)** suggested that all six genes were essential for modeling. The nomogram **(E)** was constructed based on the model. The ROC and the calibration **(D,F)** indicated the acceptable accuracy [Area Under Curve (AUC) of 3-year survival: 0.827; AUC of 5-year survival: 0.84] and discrimination of the nomogram.

We constructed the ceRNA network, which includes 28 genes based on the interactions of 13 lncRNA–miRNA pairs and 10 miRNA–mRNA pairs (Figure 3A; Table 1). We used Cox regression, Kaplan–Meier and the log-rank test to examine the relationship between the biomarkers in the bone metastasis ceRNA network and the prognosis. GAS1RR (*P* = 0.001), AXIN2 (*P* = 0.001), AC017104.1 (*P* = 0.002), RASSF8-AS1 (*P* = 0.008), CGN (*P* = 0.008), MIR4458HG (*P* = 0.011), hsa-miR-125b-5p (*P* = 0.012), linc01105 (*P* = 0.014), and CASP9 (*P* = 0.012) were found to be significant in the Kaplan–Meier analysis (Figures 3B–J). Six potential prognosis-related biomarkers were regarded as key members of the ceRNA network and were integrated into a new multivariable model (Figure 4A). The nomogram was constructed based on the model (Figure 4E). The results of the Lasso regression revealed that all six genes were essential for modeling (Figures 4B,C). Furthermore, the ROC and the calibration curves suggested an acceptable accuracy (AUC of 3-year survival: 0.827; AUC of 5-year survival: 0.840) and discrimination of the nomogram (Figures 4D,F).

**Table 1:** Hypergeometric testing and correlation analysis results of ceRNAs network.

**LncRNA**	**Protein-coding RNA**	**MiRNA**	**Correlation *P***	**Hypergeometric test *P***
DGCR11	BTBD3	hsa-miR-181c-5p	8.11E-03	6.43E-03
RASSF8-AS1	CASP9	hsa-miR-582-5p	4.25E-02	1.05E-06
MIR4458HG	LCN2	hsa-miR-138-5p	1.44E-02	9.65E-03
GAS1RR	LCN2	hsa-miR-138-5p	4.37E-03	8.04E-03
BAALC-AS1	FOXP1	hsa-miR-1-3p	1.24E-02	9.92E-06
AC017104.1	LCN2	hsa-miR-138-5p	6.24E-04	1.69E-10
LINC01105	AQP1	hsa-miR-320a	1.50E-02	6.52E-06
NR2F1-AS1	SCNN1A	hsa-miR-125b-5p	1.94E-02	1.80E-03
NR2F1-AS1	CGN	hsa-miR-125b-5p	1.94E-02	1.37E-02
NR2F1-AS1	AQP1	hsa-miR-320a	1.94E-02	4.03E-02
NR2F1-AS1	FAM162A	hsa-miR-139-5p	1.94E-02	2.05E-02
NR2F1-AS1	ATP1B1	hsa-miR-192-5p	1.94E-02	1.52E-03
NR2F1-AS1	AXIN2	hsa-miR-15b-5p,hsa-miR-16-5p	1.48E-02	4.61E-05

*ceRNAs, Competing endogenous RNAs; LncRNA, Long non-coding RNA; MiRNA, microRNA. Pearson correlation analysis was performed for lncRNA in the upstream and mRNA in the downstream, and the P value <0.05 was shown in Table*.

### The Composition of Immune Cells in MESO

**Figure 5:**
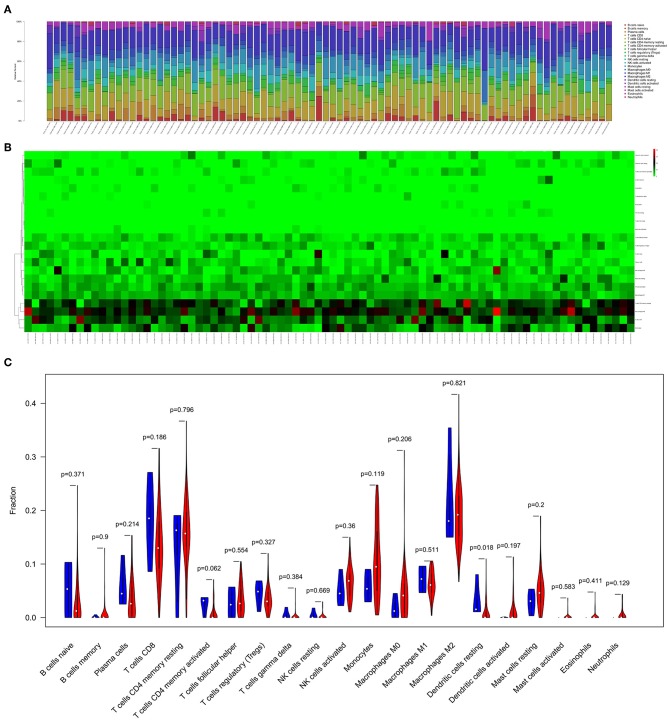
The composition **(A)** and heatmap **(B)** of immune cells estimated by CIBERSORT algorithm in mesothelioma. The violin plot of immune cells **(C)** and the blue and red bar represent recurrent the tumor group and primary tumor group, respectively.

Figures 5A,B displayed the proportion of 22 immune cells detected by the CIBERSORT algorithm. The violin plot (Figure 5C) depicted results of the Wilcoxon rank-sum test, which showed that the fraction of dendritic cells resting (*P* = 0.018) was significantly different between the bone metastasis group and the non-bone metastasis group.

### Clinic Correlation and Nomogram of Immune Cells

**Figure 6:**
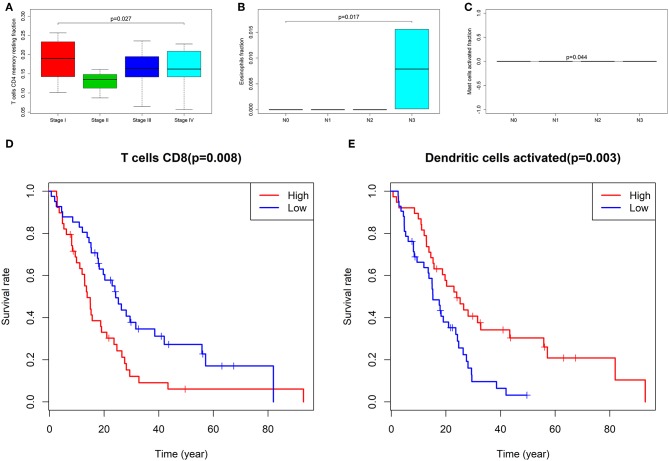
The fraction of T cells CD4 memory resting between four stages of cancer **(A)**; the fraction of eosinophils **(B)** and Mast cells activated **(C)** between T groups; the Kaplan–Meier survival curves of Fraction of T cells CD8 **(D)** and Dendritic cells activated **(E)**.

We used the non-parameter test and Kaplan–Meier survival analysis to examine the association between the fraction of different immune cell subtypes and the prognosis. The fraction of T cells CD4 memory resting was significantly different among four tumor stages of cancer (*P* = 0.027, Figure 6A). The fraction of eosinophils (*P* = 0.017, Figure 6B) and mast cells activated (*P* = 0.044, Figure 6C) was significantly different among T stages. The fraction of T cells CD8 (*P* = 0.080, Figure 6D) and Dendritic cells activated (*P* = 0.003) was found to be significantly associated with overall survival (Figure 6E).

**Figure 7:**
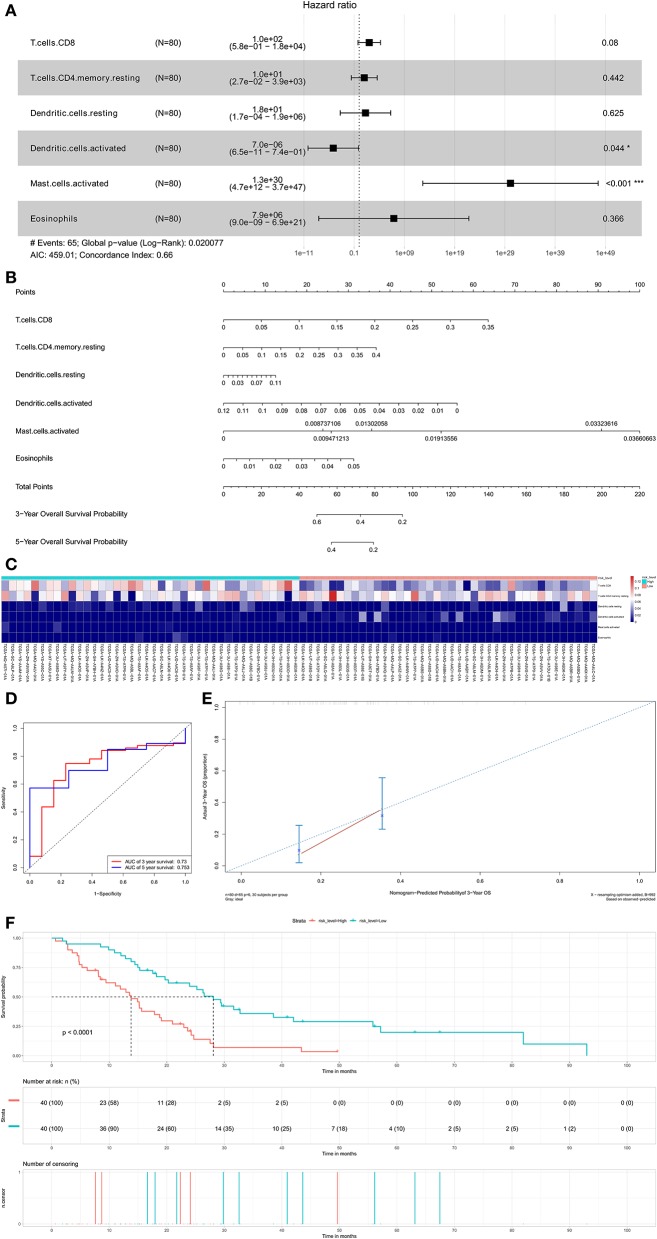
The results of the multivariate Cox regression **(A)** based on prognosis-related immune cells nomogram **(B)**; the ROC curves **(C)**; the heatmap of the six immune cells in Cox regression model **(D)**; Nomogram-Predicted probabilitu of 3-year overall survival **(E)**; the Kaplan–Meier survival curve **(F)**.

Six potential prognosis-related biomarkers were regarded as key members among 22 immune cell subtypes and were integrated into a new multivariable model (Figure 7A). The nomogram was constructed based on this model (Figure 7B). All the cases were identified as high or low risk groups according to the nomogram model. The proportion of immune cells and the survival of each group are depicted in Figure 7C. The ROC and the calibration curve demonstrated the nomogram's good accuracy (AUC of 3-year survival: 0.730; AUC of 5-year survival: 0.753) and concordance (Figures 7D,E). The result of Kaplan–Meier analysis showed the significant difference between the high and low risk group (Figure 7F).

### The Co-expression Analysis

**Figure 8:**
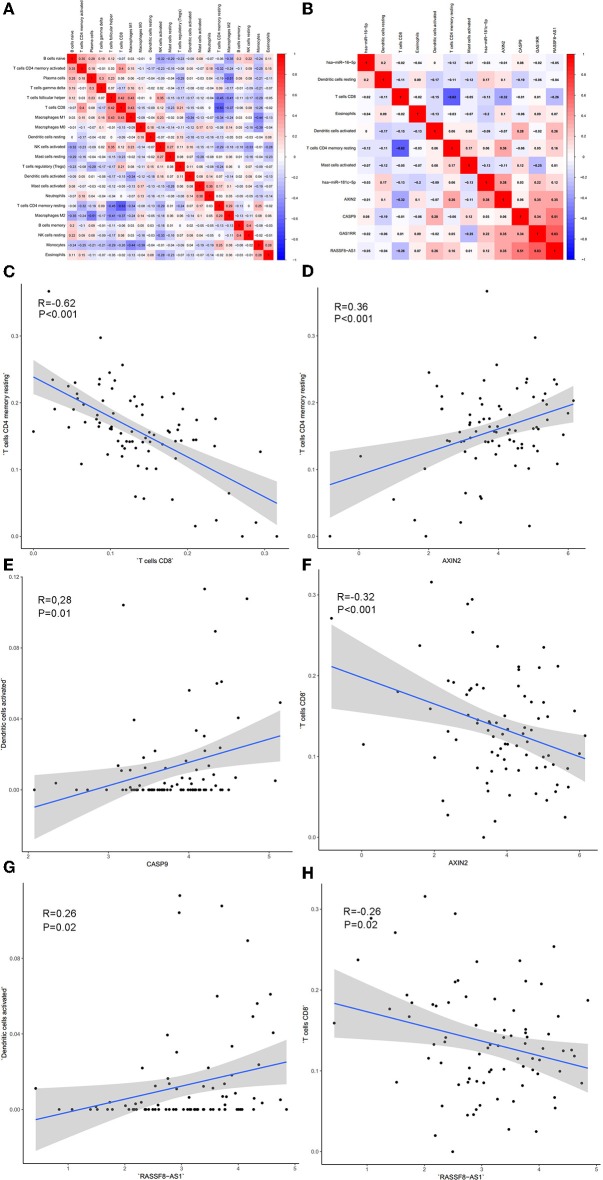
The co-expression patterns among fractions of immune cells **(A)**; the co-expression patterns among fractions of immune cells and key members in the ceRNA network **(B)**; the relationships among immune cells and between ceRNAs and immune cells were calculated using Pearson correlation coefficients: T cells CD4 memory resting and T cells CD4 **(C)**; T cell CD4 memory resting and AXIN2 **(D)**; Dendritic cells activated and CASP9 **(E)**; T cells CD8 and AXIN2 **(F)**; Dendritic cells activated and RASSF8-AS1 **(G)**; T cells CD8 and RASSF8-AS1 **(H)**.

Significant co-expression patterns between proportions of immune cells (Figure 8A) and ceRNA-immune cells (Figure 8B) were analyzed via Pearson correlation analysis. The fraction of T cells CD4 memory resting is negatively associated with CD8 T cells (*R* = −0.62, *P* < 0.001) and positively associated with AXIN2 expression (*R* = 0.36, *P* < 0.001). The fraction of dendritic cells activated is positively associated with the expression of CASP9 (*R* = 0.28, *P* = 0.010) and RASSF8-AS1 (*R* = 0.26, *P* = 0.020). The fraction of T cells CD8 is negatively associated with the expression of AXIN2 (*R* = −0.32, *P* < 0.001) and RASSF8-AS1 (*R* = −0.26, *P* = 0.020) (Figures 8C–H).

## Discussion

Malignant mesothelioma, deriving from mesothelial cells, is a tumor type with aggressive invasion and poor prognosis. Distant metastasis, especially for bone metastasis, is often found at the late stage, but its molecular mechanism is still unclear. During tumor initiation and metastasis, molecular, and cellular characteristics play a crucial part and are regarded as prognostic factors (Rodina et al., 2016). Among them, ceRNAs network and tumor-infiltrating immune cells attract our attention. In this study, we first discovered the significant tumor-infiltrating immune cells and ceRNAs between primary mesothelioma and bone metastasis and established two prediction models. The high AUC values of both nomograms proved their clinical application.

The ceRNA networks consist of protein-coding mRNAs and ncRNAs, such as miRNAs and lncRNAs (Salmena et al., 2011). In the present study, we utilized bioinformatics analysis to identify the ceRNA networks that regulate bone metastases from mesothelioma with 10 protein-coding mRNAs, 8 lncRNAs, and 10 miRNAs. In the ceRNA networks, GAS1RR, AXIN2, AC017104.1, RASSF8-AS1, CGN, MIR4458HG, has-miR-125b-5p, LINC01105, and CASP9 were significantly associated with overall survival in mesothelioma. The prediction nomogram was constructed and the AUC value of 3-year survival and 5-year survival was 0.827 and 0.840, respectively.

According to the hypergeometric testing and correlation analysis, the results of the ceRNAs network revealed that hsa-miR-582-5p (miRNA), CASP9 (protein-coding RNA), and RASSF8-AS1 (LncRNA) were correlated (*P* = 0.486). Subsequently, the correlation analysis between ceRNAs and tumor-infiltrating immune cells discovered that both CASP9 and RASSF8-AS1 were positively correlated with Dendritic cells activated. Thus, we focused on CASP9 and RASSF8-AS1 in our study.

RASSF8-AS1 is an endogenous, unspliced long noncoding RNA (lncRNA) transcribed from the opposite strand of the RASSF8 gene. Divergent lncRNAs can perform biological processes related to the protein through the regulation of the transcription of adjacent protein-coding genes, which is a widely existing new model of gene expression regulation (Luo et al., 2016). RASSF8 is widely expressed in all major organs and tissues, and endogenous RASSF8 is expressed in both the cell membrane and the nucleus. It promotes cell–cell adhesion by maintaining the stability of the adhesion junction (Lock et al., 2010). Zhang et al. discovered that RASSF8 downregulation promoted lymph angiogenesis and metastasis in esophageal squamous-cell carcinomas. Tumor cells with low RASSF8 expression had higher migratory ability and promoted lymph angiogenesis both *in vitro* and *in vivo* (Zhang et al., 2015). They were also associated with tumorigenesis and metastasis in gastric cancer and malignant thyroid neoplasms (Li et al., 2013; He et al., 2017). RASSF8-AS1 may be involve in mesothelioma metastasis through the downregulation of RASSF8 as a cis-acting element. CASP9 is thought to play a central role in apoptosis and to be a tumor suppressor. Dendritic cells (DCs), known as major antigen-presenting cells, can initiate and direct adaptive immune responses with their surface expression of pattern recognition receptors. DC vaccination acts as a promising approach to further promote cancer immunotherapy. However, some studies demonstrated that cancer cells might still influence DCs to improve an immunosuppressive phenotype. It has been reported that pDCs could recruit other immunosuppressive immune cells comprising myeloid-derived suppressor cells (MDSCs) and Tregs, thereby promoting tumor progression and metastasis (Shurin et al., 2011).

MiRNAs are able to bind to the 3′ untranslated region (3′ UTR) of the target mRNAs in a complementary base-pairing manner, which has been demonstrated to contribute to the suppression and induction of oncogenesis (Huang et al., 2014). It has been reported that miR-582-5p interacted with the CASP9 mRNA 3′ UTR and induced a downregulation of CASP9 expression, which was consistent with our present study (Floyd et al., 2014). One of the main functions of IncRNAs, indirectly regulating mRNA, DNA, and protein expressions, was implemented through miRNA binding to silence the miRNA. The targeting relationship between hsa-mir-582-5p and RASSF8-AS1 was also verified by IP experiments in another study (Kameswaran et al., 2014). We inferred that the mechanism of hsa-miR-582-5p regulating CASP9 and dendritic cells resting might play a critical role in bone metastasis in the correlation analysis and RASSF8-AS1 might indirectly regulate CASP9 through interaction with hsa-miR-582-5p.

We also found T cells CD8, T cells CD4 memory resting and Axin2 were all associated with metastasis prognosis. The proportion of T cells CD8 and expression of Axin2 were found to be significantly associated with overall survival. The proportion of T cells CD4 memory resting was significantly different among four tumor stages of cancer. Moreover, co-expression shows that the fraction of T cells CD8 is negatively associated with the expression of AXIN2. The fraction of T cells CD4 memory resting is negatively associated with CD8 T cells and positively associated with AXIN2 expression. Thus, we presumed that the mechanism of AXIN2 regulating T cells CD8 and T cells CD4 memory resting might have an influence on tumor progression and metastasis.

Axin2 is a protein coding gene, identified as a regulator of the Wnt signaling pathway (Zeng et al., 1997). Axin2 can form a compound with adenomatous polyposis coli (APC) and glycogen synthase kinase-3β (GSK-3β) and then negatively regulate the Wnt/β-catenin signaling pathway, which has been considered as one of the primary pathways participates in cell proliferation, differentiation and migration (Ikeda et al., 1998; Sharpe et al., 2001). Axin2 inhibits the translocation of β-catenin into the nucleus to inhibit it from subsequently binding to transcription factors, and it also downregulates several target genes such as the vascular endothelial growth factor (VEGF), cyclin D1 and matrix metalloproteinases (MMP) (Cadigan and Nusse, 1997; Tortelote et al., 2017). In this way, axin2 is identified as a tumor suppressor (Behrens et al., 1998).

Previous study indicated that WNT activation downregulates the proportion of T cells memory by decreasing the critical transcription factor for the generation of T cells memory (Shen et al., 2013). That is consistent with our result that AXIN2 is positively associated with CD4 T cells memory resting. The WNT/β-catenin pathway also has an impact on thymocyte development, especially in the double-negative to double-positive transition and positive/negative selection progress (Pongracz et al., 2006; Yu et al., 2007; Kovalovsky et al., 2009; Shen et al., 2013). Yu et al. confirmed the expression of stabilized β-catenin accelerated the production of CD8 single-positive thymocytes using a transgenic mouse strain (Yu et al., 2007), which indicated that AXIN2 may be negatively associated with CD8 T cells, and our study indeed proved that. Thus, we inferred that the WNT/β-catenin pathway may be the potential mechanism behind the association between AXIN2 and the fraction of immune cells.

In order to explore the gene and protein expressions of key biomarkers in the mesothelioma, normal tissue and cell lines we performed a dimensional validation (Table 2), applying multiple databases. First of all, across 17 analyses, AXIN2 (Median rank 178, *P* < 0.05) was highly expressed in various tumors compared to normal tissue, while CASP9 (Median rank 28, *P* < 0.05) showed higher expression only in medulloblastoma in the Oncomine (Figure S1). The integrative analysis of genomics and the clinical profiles using the cBioPortal suggested that AXIN2, CASP9, CGN, RASSF8-AS1, and MIR4458HG were highly expressed in mesothelioma compared to some other types of cancer (Figure S2). At the cellular level, the expression of ceRNA biomarkers was detected in various mesothelioma cell lines using the CCLE database (Figure S3). In clinical analysis, high expression of CGN showed higher survival probability in the UALCAN database (Figure S6), and high expression of AXIN2, CASP9, and CGN indicated higher overall survival in the PROGgeneV2 database (Figure S4). What's more, AXIN2, CASP9, and CGN had a significant Protein-Protein interaction network, according to the STRING database (Figure S5).

**Table 2:** Summary of multidimensional external validation results based on multiple databases.

**Database**	**AXIN2**		**CASP9**		**CGN**		**RASSF8-AS1**		**MIR4458HG**		**Results**
	**Cancer**	**Normal**	**Cancer**	**Normal**	**Cancer**	**Normal**	**Cancer**	**Normal**	**Cancer**	**Normal**	
Oncomine	↑	↓	↑	↓	-	-	-	-	-	-	According to 17 analyses, AXIN was highly expressed in various tumors compared to normal tissue, while CASP9 was highly expressed only in medulloblastoma across two analyses (Figure S1).
cBioPortal	↑	↓	↑	↓	↑	↓	↑	↓	↑	↓	AXIN2, CASP9, CGN, RASSF8-AS1, and MIR4458HG were highly expressed in mesothelioma compared to some other types of cancer (Figure S2).
CCLE	-	-	-	-	-	-	-	-	-	-	AXIN2, CASP9, CGN, RASSF8-AS1, and MIR4458HG were expressed in various mesothelioma cell lines but not in immune cells biomarkers (Figure S3).
UALCAN	-	-	-	-	-	-	-	-	-	-	High expression of CGN indicated higher survival probability (Figure S6).
PROGgeneV2	-	-	-	-	-	-	-	-	-	-	High expression of AXIN2, CASP9, and CGN indicated higher overall survival (Figure S4).
STRING	-	-	-	-	-	-	-	-	-	-	AXIN2, CASP9, and CGN had significant Protein-Protein interaction network (Figure S5).

“*↑” was defined as a significantly upregulated gene; “↓” was defined as a significantly downregulated gene; “-” was defined as a gene with no significant difference in expression. CCLE, Cancer Cell Line Encyclopedia*.

There are inevitably several limitations to our study that should be acknowledged. All data series in our study were from Western countries; therefore, one should be cautious in applying this conclusion to patients from Asian countries. Due to the limited amount of data available in public datasets, the clinicopathological parameters analyzed in this study are not comprehensive, which may cause analysis deviation. Thus, we explored multiple databases to observe gene and protein expression of key biomarkers at the cellular and tissue levels to minimize bias. The results showed that key biomarkers in our nomogram model were significantly changed in mesothelioma (Figures S1–S6). But, ignoring these limitations, our study first applied the nomograms model to predict prognoses of mesothelioma patients on the basis of ceRNA networks and tumor-infiltrating immune cells. We also inferred that AXIN2 probably regulates the cell differentiation of T cells CD8 and T cells CD4 memory resting through the WNT pathway, subsequently influencing cancer prognoses.

The tumor microenvironment often affects the invasive processes. The extracellular matrix molecules and secreted growth factors are involved in the transition of tumor cells into an invasive phenotype. It is noteworthy that the invasion and metastasis of tumor cells may have nothing to do with the proliferation of tumor cells but have occurred already at the early developmental stage of the tumor (Hosseini et al., 2016). Therefore, it is essential to identify molecules that may lead to mesothelioma invasion and metastasis. We established two nomograms to predict survival and bone metastasis of mesothelioma patients based on tumor-infiltrating immune cells and ceRNA networks, and we determined their utility by the high AUC values. The prediction nomograms proposed in this study might provide further comprehensive clinical information for furthering the personalized management of mesothelioma patients. Our study is a correlation study from multiple dimensions rather than a biological mechanism study. Based on the results of this study, we will conduct biological experiments to further validate our conclusion. A luciferase reporter assay will be using to prove the direct interaction mechanism of ceRNAs and Chromatin Immunoprecipitation (ChIP) to verify the molecular expression level of protein product. Next, we would like to demonstrate a molecular crosstalk between cancer cells and immune cells. We wish to consider if an exosome secreted by tumor cells contains ceRNAs, which act on immune cells and mediate the metastasis of Mesothelioma.

## Data Availability Statement

Publicly available datasets were analyzed in this study. This data can be found here: The relevant data provided by TCGA (https://tcga-data.nci.nih.gov/tcga/).

## Author Contributions

TM, ZH, and JZ conceived and designed the study. RH performed the bioinformatic analysis. GW, DS, and PY performed the data interpretation. JW and ZZ wrote the manuscript. HY and PH conducted the literature search. XZ, HW, and QL contributed to the revision of the manuscript draft. All authors read and approved the final manuscript.

### Conflict of Interest

The authors declare that the research was conducted in the absence of any commercial or financial relationships that could be construed as a potential conflict of interest.

## Supplementary Material

Figure S1 Validation of AXIN2 **(A,B)** and CASP9 **(C,D)** on a transcriptional level in multiple cancer types and multiple studies using the Oncomine database.

Figure S2 Integrative analysis of genomics using the cBioPortal suggested.

Figure S3 Expression of ceRNA biomarkers in mesothelioma cell lines using CCLE database.

Figure S4 Validation of expression levels of AXIN2 **(A)**, CASP9 **(B)**, and CGN **(C)** with overall survival of mesothelioma patients using P.

Figure S5 Protein-Protein interaction network (PPI) of AXIN2 **(A)**, CASP9 **(B)**, and CGN **(C)** using the String database.

Figure S6 Validation of CGN expression level on mesothelioma patient survival using the ULCAN database.

Table S1 Baseline information of 87 patients diagnosed with mesothelioma, available from the TCGA. SD, Standard deviation.

## Abbreviations

TIIC: Tumor infiltrates immune cells
ceRNAs: Competing endogenous RNAs
LncRNA: Long non-coding RNA
MiRNA: microRNA CCLE: Cancer Cell Line Encyclopedia
SD: Standard deviation.
