# Supplementary figures and images for: The Construction and Analysis of ceRNA Network and Patterns of Immune Infiltration in Mesothelioma With Bone Metastasis
Source: Front Bioeng Biotechnol. 2019 Oct 18;7:257. doi: 10.3389/fbioe.2019.00257 (PMC6813567; doi:10.3389/fbioe.2019.00257)

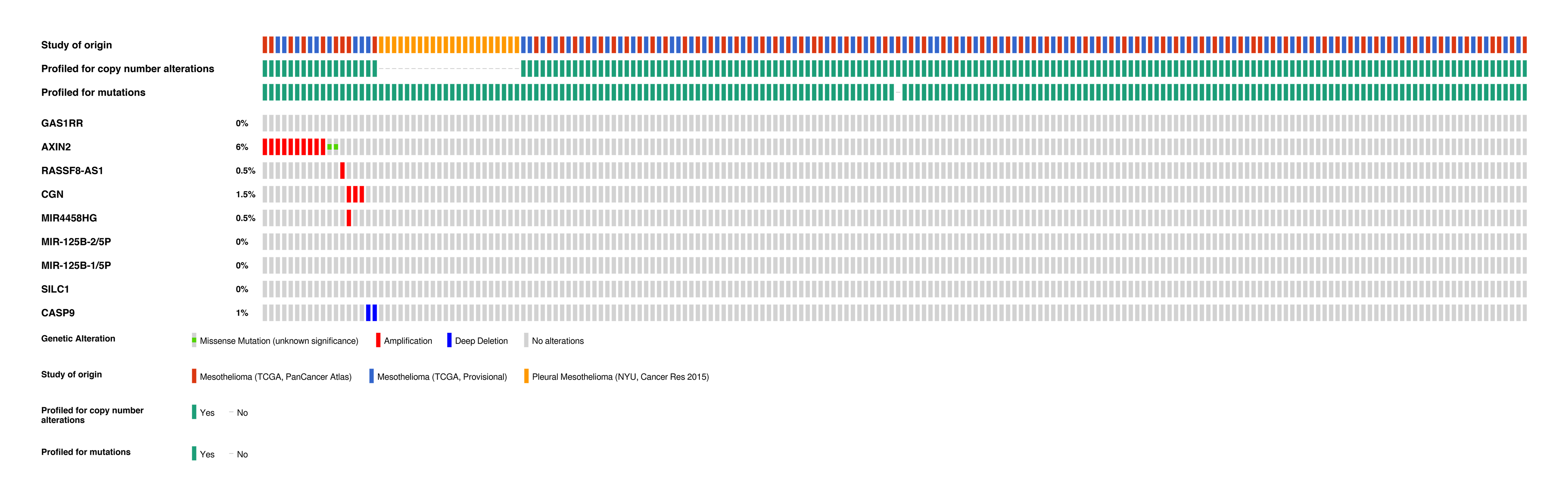

Supplement: Figure S2 — Integrative analysis of genomics using the cBioPortal suggested. [file Image_2.TIF]

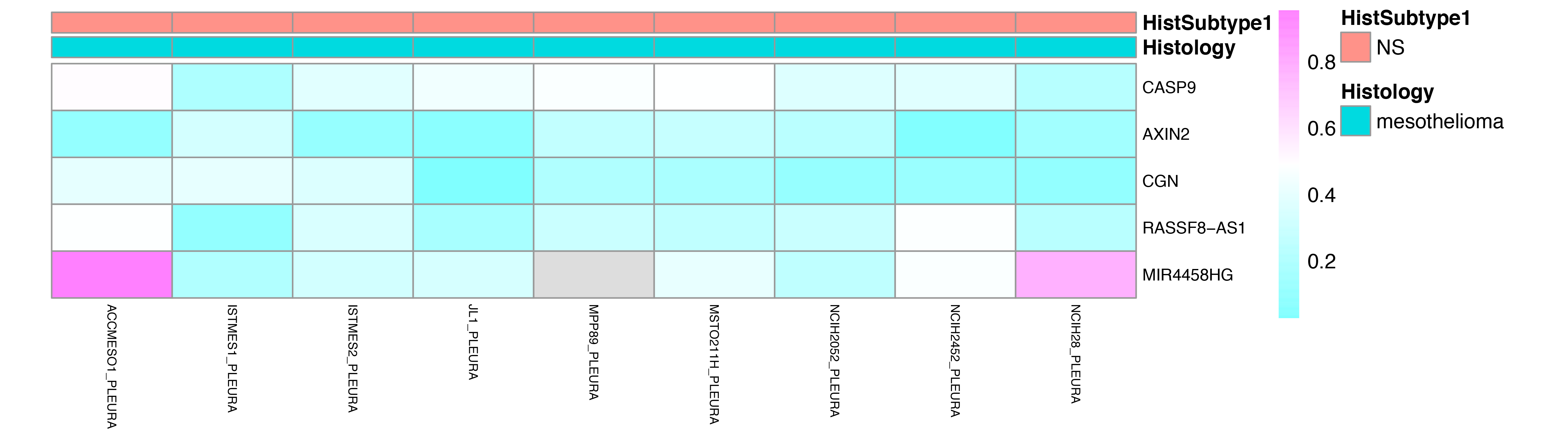

Supplement: Figure S3 — Expression of ceRNA biomarkers in mesothelioma cell lines using CCLE database. [file Image_3.TIF]

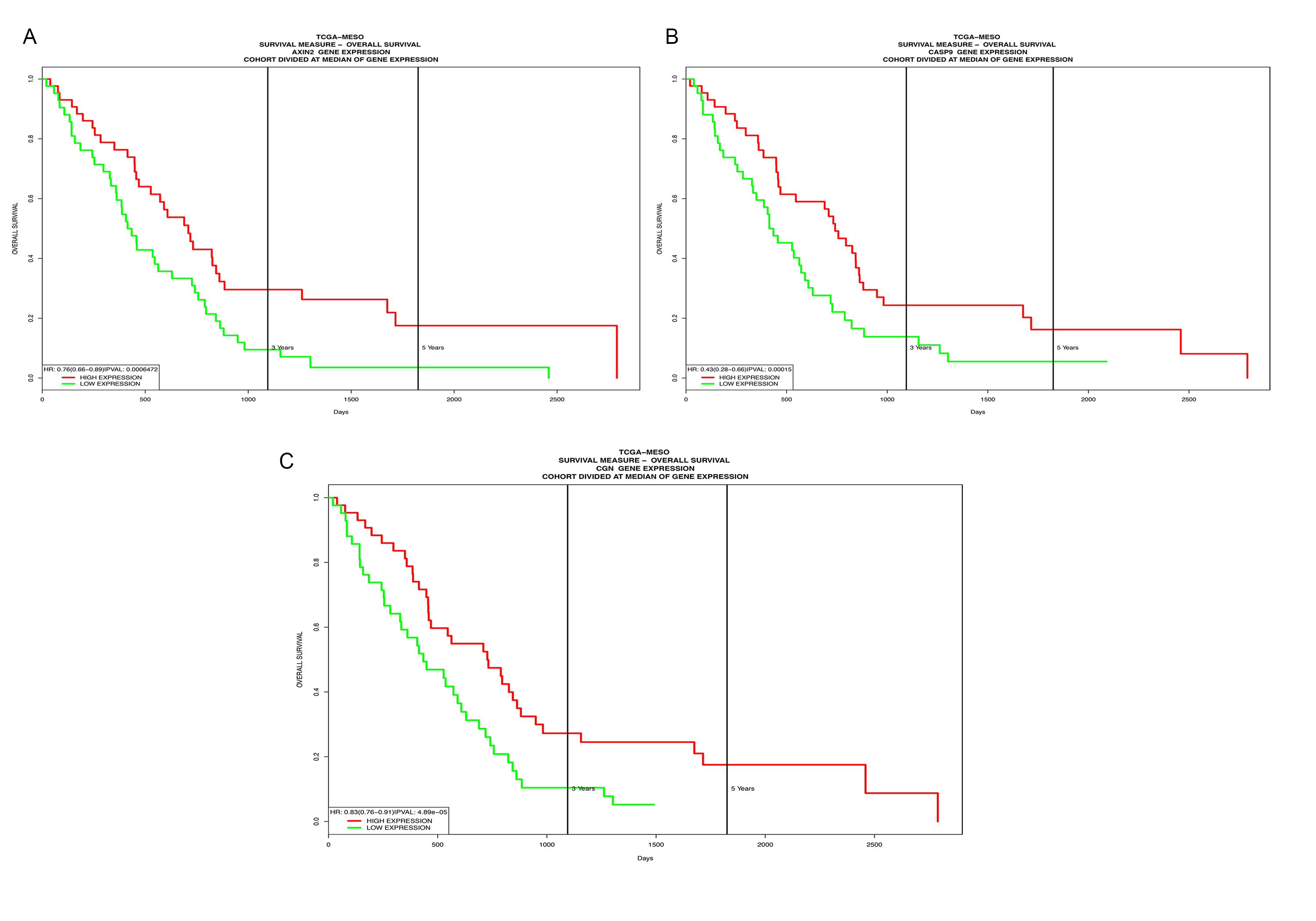

Supplement: Figure S4 — Validation of expression levels of AXIN2 (A), CASP9 (B), and CGN (C) with overall survival of mesothelioma patients using P. [file Image_4.TIF]

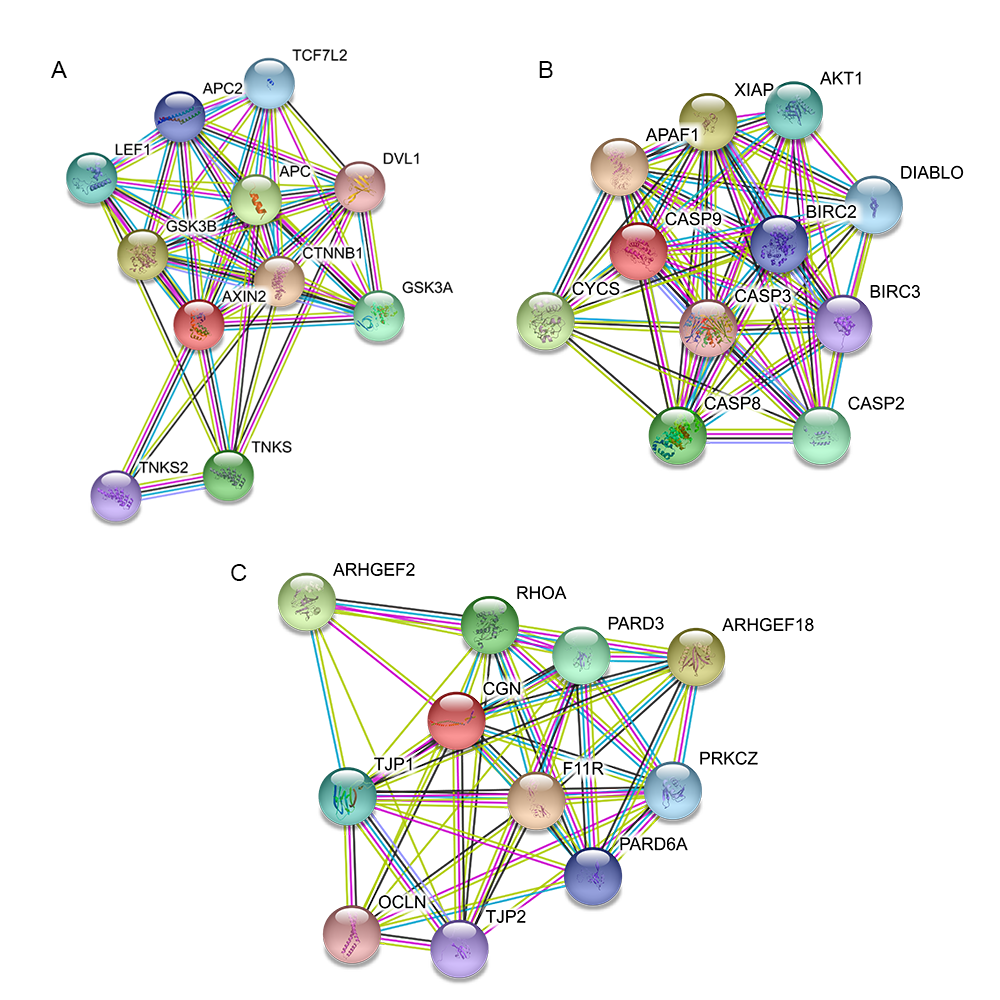

Supplement: Figure S5 — Protein-Protein interaction network (PPI) of AXIN2 (A), CASP9 (B), and CGN (C) using the String database. [file Image_5.TIF]

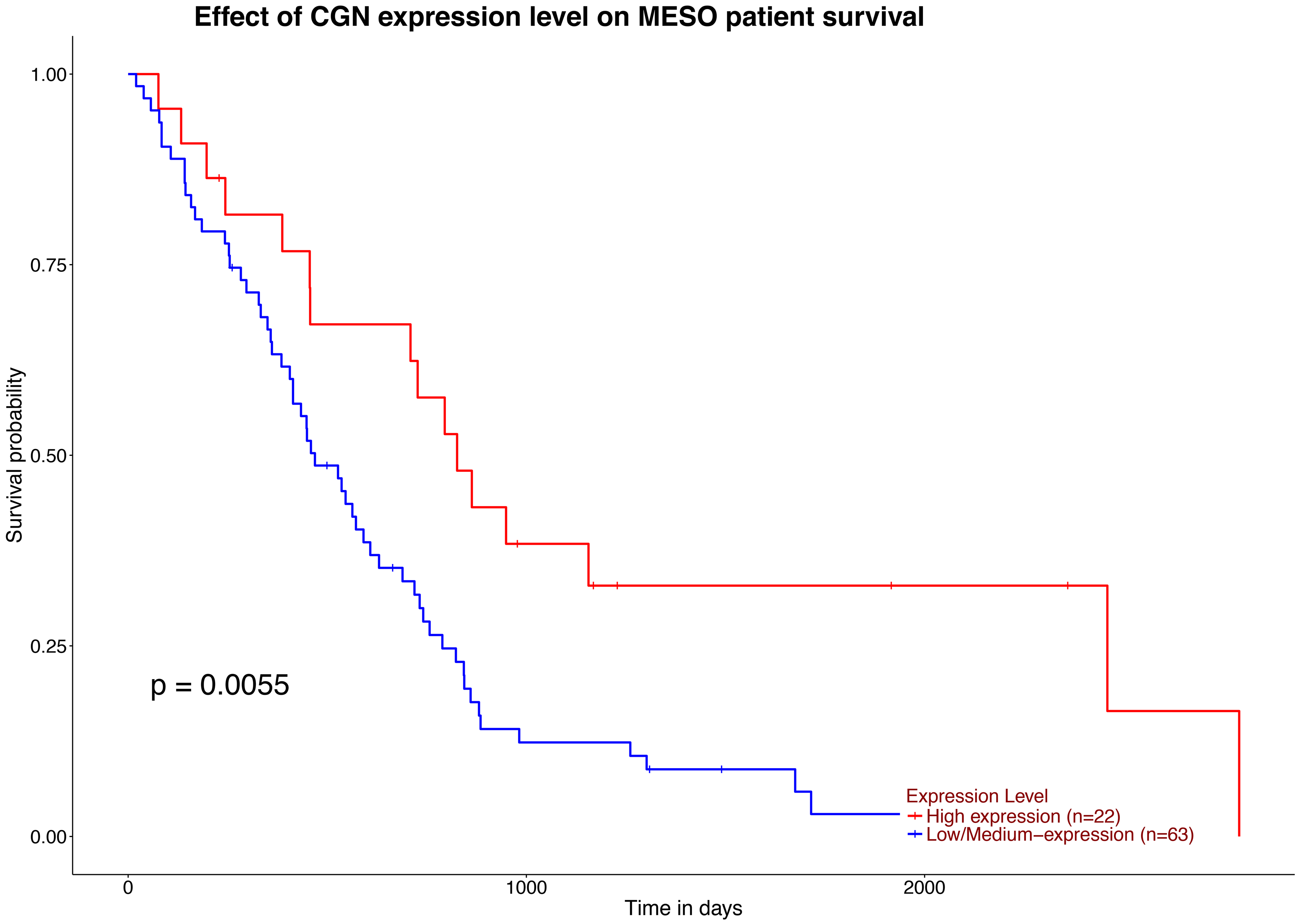

Supplement: Figure S6 — Validation of CGN expression level on mesothelioma patient survival using the ULCAN database. [file Image_6.TIF]
